# Reverse iontophoresis of urea in health and chronic kidney disease: a potential diagnostic and monitoring tool?

**DOI:** 10.1111/j.1365-2362.2012.02657.x

**Published:** 2012-08

**Authors:** Leonard M Ebah, Ian Read, Andrew Sayce, Jane Morgan, Christopher Chaloner, Paul Brenchley, Sandip Mitra

**Affiliations:** * Department of Renal Medicine and Renal Research, Manchester Royal InfirmaryManchester, UK; † School of Biomedicine, University of ManchesterManchester, UK; ‡ Department of Clinical Biochemistry, Manchester Royal InfirmaryManchester, UK

**Keywords:** Chronic kidney disease, dialysis, iontophoresis, urea

## Abstract

**Background:**

Patients with chronic kidney disease (CKD) need regular monitoring, usually by blood urea and creatinine measurements, needing venepuncture, frequent attendances and a healthcare professional, with significant inconvenience. Noninvasive monitoring will potentially simplify and improve monitoring. We tested the potential of transdermal reverse iontophoresis of urea in patients with CKD and healthy controls.

**Methods:**

Using a MIC 2® Iontophoresis Controller, reverse iontophoresis was applied on the forearm of five healthy subjects (controls) and 18 patients with CKD for 3–5 h. Urea extracted at the cathode was measured and compared with plasma urea.

**Results:**

Reverse iontophoresis at 250 μA was entirely safe for the duration. Cathodal buffer urea linearly correlated with plasma urea after 2 h (*r* = 0·82, *P* < 0·0001), to 3·5 h current application (*r* = 0·89, *P* = 0·007). The linear equations *y* = 0·24*x* + 1 and *y* = 0·21*x* + 4·63 predicted plasma urea (*y*) from cathodal urea after 2 and 3 h, respectively. Cathodal urea concentration in controls was significantly lower than in patients with CKD after a minimum current application of 2 h (*P* < 0·0001), with the separation between the two groups becoming more apparent with longer application (*P* = 0·003). A cathodal urea cut-off of 30 μM gave a sensitivity of 83·3% and positive predictive value of 87% CKD. During haemodialysis, the fall in cathodal urea was able to track that of blood urea.

**Conclusion:**

Reverse iontophoresis is safe, can potentially discriminate patients with CKD and healthy subjects and is able to track blood urea changes on dialysis. Further development of the technology for routine use can lead to an exciting opportunity for its use in diagnostics and monitoring.

## Introduction

The global burden of chronic kidney disease (CKD) is increasing, due to a number of factors such as the pandemics of diabetes and obesity, but partly due to improved screening [1,2]. This has led to a huge demand on resources for the diagnosis of new cases and for monitoring those already diagnosed with CKD [3]. Currently, diagnosis and monitoring are carried out mainly by measuring blood urea and creatinine, both small water-soluble uraemic retention solutes [4–6]. This requires frequent blood sampling, necessitating the presence of a trained healthcare professional and causing significant pain, discomfort and inconvenience for the patient. In the latter stages of CKD, weekly attendances may be necessary as the need to commence dialysis may not be otherwise predictable [6]. For those already on dialysis, frequent monitoring of blood urea is also performed to determine the efficacy of the treatment [5]. This requires the attendance of a healthcare professional or necessitates an outpatient clinic visit, particularly cumbersome for patients in the community such as those on home-based therapies or with a functioning transplant. The advent of a sensitive noninvasive method of monitoring progression of CKD and dialysis treatment could simplify monitoring both from a service and user perspective.

Some investigators have trialled noninvasive approaches to monitoring kidney disease severity and efficacy of dialysis treatment. Narasimhan *et al.* [7] showed a good correlation between breath ammonia levels and blood urea and creatinine during haemodialysis. Other approaches have been trialled to monitor kidney disease progression or dialysis, but none is in routine clinical use. Through a small current applied on the skin, reverse iontophoresis extracts small charged species like potassium by ‘electromigration’ to the electrode of opposing charge and neutral polar or zwitterionic compounds like urea and glucose by ‘electrosmosis’ [8]. Charged drugs like lithium [9] and phenytoin [10] have been successfully monitored by transdermal reverse iontophoresis. The Glucowatch® Biographer was the first commercial product that employed this technique to noninvasively monitor an endogenous substance (glucose) [11–13]. Glucose thus extracted by electrosmosis correlated with blood glucose levels [12]. This renewed enthusiasm in the use of reverse iontophoresis. Wascotte *et al.* [14] showed both *in vitro* and *in vivo* [15] that iontophoretically extracted urea may segregate healthy volunteers and patients with CKD, after applying a 0·8 mA current on the skin for 2 h. In steady-state conditions, subdermal urea concentrations are in equilibrium with plasma urea in both patients with CKD and healthy individuals [16,17]. Hence, extracted subdermal interstitial urea could be considered to reflect blood urea. This has opened the possibility of noninvasive transdermal diagnosis and monitoring of CKD. However, to date, few *in vivo* studies have pursued this theme. Furthermore, although linearity between extracted urea and blood urea only became discernible beyond 1 h, previous studies have been limited to up to 2 h of current application. This potentially leaves a very short window for attaining optimal linearity with blood urea.

Other uraemic retention solutes like potassium [14] and homocysteine [18] have been shown in *in vitro* models to reliably track subdermal levels, adding further weight to the possible utility of this technique in CKD. This study aimed to determine whether reverse iontophoresis applied over a longer period (3–5 h) (a) was safe and well tolerated, (b) could be used to predict blood urea levels accurately in healthy volunteers and patients with CKD, (c) would yield iontophoretic fluxes that can segregate healthy volunteers and patients with CKD in steady states and (d) whether iontophoretically extracted urea reliably tracks rapid changes in urea levels during haemodialysis (HD).

## Methods

### Recruitment

The study was given ethical approval by the National Health Service Research Ethics Committee. Eligible study subjects were invited to attend a 3–5-h research session that took place in a dedicated temperature-controlled research room at the Wellcome Trust Clinical Research Facility, Manchester and on the Renal Unit at Manchester Royal Infirmary between January 2009 and July 2011. All subjects gave written informed consent, and studies were carried out in accordance with the Helsinki declaration. Healthy volunteers, serving as unmatched controls, and patients with advanced CKD (not yet on dialysis) or CKD on dialysis were recruited. Subjects with any type of skin condition or uraemic xerostosis and those with a pacemaker or implantable defibrillator were excluded.

### Reverse iontophoresis

**Figure 1:**
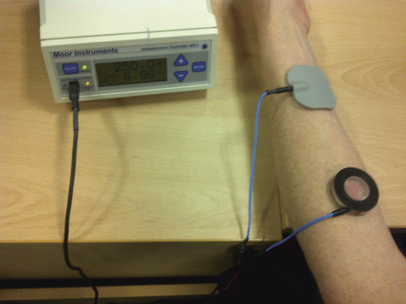
The reverse iontophoresis set-up. The iontophoresis controller delivers a constant current of 250 μA between the two electrodes pictured. The cathode chamber was filled with 1·5 mL fresh buffer that was sampled every 30 min.

Reverse iontophoresis was performed on the nondominant arm, or the fistula-free arm for those with an arterio-venous fistula. Sampling was performed during dialysis for the haemodialysis patients. Peritoneal dialysis patients were drained dry prior to studies. A MIC 2® Iontophoresis Controller (Moor Instruments Inc., Wilmington, DE, USA) was used to deliver a constant current of 250 μA between two electrodes located about 10 cm apart on the ventral forearm that had been pre-cleaned and degreased with 70% ethanol. The anode was an inactive gel electrode, and the cathode was made of 1 mM TRIS (hydroxymethylaminomethane) buffer at pH 8·75 at 37 °C contained in a 1·5-mL chamber. The buffer was in contact with the skin over a 3·8-cm^2^ area. Once the controller was switched on, it delivered a potential difference depending on skin resistance to maintain the current constant at 250 μA (Fig. 1). After 30 min, the controller switched off automatically and the cathode chamber buffer (the sample) was emptied into an Eppendorf vial. The skin was dried, and the cycle was repeated at least five times for each subject and for the duration of dialysis in HD patients. Whenever possible, one ‘run’ was performed before the commencement of dialysis (pre-dialysis sample) and after the end of dialysis (post-dialysis sample). During HD, blood was sampled every 30 min from the arterial port. In others, one blood sample was obtained by venepuncture at the end of the session. Blood samples were collected into EDTA tubes, centrifuged at 770 *g* for 10 min, plasma aliquoted and all samples stored at −80 °C until analysis. Any pain, discomfort, skin irritation or lesion as a result of iontophoretic current application were noted, as well as its duration and severity (by means of a 0–10 analogue pain score and a skin irritation chart, respectively).

### Sample analysis

Plasma urea was measured using a colorimetric urease and glutamate dehydrogenase method with a lower detection limit of 0·5 mM (Cobas®; Roche® Diagnostics GmbH, and Mannheim, Germany). As the urea concentrations in the iontophoretic buffer were much smaller (of the micromolar order), this required a much more sensitive assay. We adapted the diacetylmonoxime method of urea assay described by Friedman [19]. Briefly, 60 μL of sample was mixed with 180 μL of colour reagent comprised of 20 mL acid reagent (190 mL distilled water, 300 mL 85% phosphoric acid and 10 mL concentrated sulphuric acid), 1000 μL 2·5% diacetylmonoxime and 400 μL 0·25% thiosemicarbazide. All reagents were of analytical grade. The plate was then sealed with PCR film, incubated for 25 min at 99 °C, cooled to 15 °C and held for 5–10 min, then transferred to an Immulon® 2HB 96-well plate and read immediately at 527 nm. All samples (*n* = 168) were analysed in triplicate and the intra-assay reproducibility thus determined, whilst a random selection of 28 of the 168 samples was re-analysed to determine the inter-assay variability.

### Statistical analysis

Statistical analysis was performed using the GraphPad Prism® Software version 5.0 (GraphPad Software Inc., La Jolla, CA USA). Results were expressed as mean and standard deviation of two or more replicates, with 95% confidence intervals where appropriate. Student's *t* test (for normally distributed data) and Mann–Whitney test (for nonnormally distributed data) were used to compare two groups. Inter-group relationship was tested using Pearson's correlation coefficient (or Spearman's if data were not normally distributed). Sensitivity analyses were performed to test the potential of reverse iontophoresis as a diagnostic or screening tool. A probability of 0·05 or less was used as a cut-off to reject the null hypothesis.

Reporting this study conforms to the Strengthening the Reporting of Observational Studies in Epidemiology Initiative (STROBE) checklist [20], and the Enhancing the Quality and Transparency of Health Research (EQUATOR) guidance [21].

## Results

### Study subjects

Twenty-three consecutive subjects were recruited in total, including 18 patients with CKD (estimated glomerular filtration rate by the Modification of Diet in Renal Disease four variable (MDRD4) formula [22], eGFR <30 mL/min/1·73 m^2^) and five healthy volunteers. The patients with CKD included six HD patients, six peritoneal dialysis (PD) and six advanced CKD patients not yet on dialysis (pre-dialysis). HD is an intermittent clearance of urea whilst PD (via dialysis) and CKD pre-dialysis (via the residual renal function) are a continuous mode of urea clearance. For purposes of analysis, the latter two categories have been grouped together as non-HD CKD patients. The baseline characteristics of study participants are summarised in Table 1.

**Table 1:** Baseline characteristics of study subjects

Category	Controls	Non-HD CKD patients	HD patients
Mean age (years)	40·6 ± 14	60·8 ± 12	55·6 ± 22
Gender	1M, 4F	5M, 7F	5M, 1F
Mean eGFR (ml/min/1·73 m^2^)	>60	11 ± 5	5 ± 1

CKD, chronic kidney disease; HD, haemodialysis.

The healthy controls were significantly younger than the patients with CKD (*P* = 0·04). All controls had normal renal function (eGFR>60). Although the non-HD CKD patients had a higher eGFR than the HD patients (*P* = 0·004), this was because of the higher eGFRs of the pre-dialysis patients in this group (mean eGFR 15·1 ± 4 vs. 6·8 ± 3 for PD patients; *P* = 0·002).

### Safety and tolerability of reverse iontophoresis

Most of the study subjects reported a mild tingling at the beginning of current application. This disappeared after a few minutes. All subjects also noted mild skin reddening where the electrodes were applied, but this also completely disappeared within a few minutes of the end of reverse iontophoresis. No burns or lasting skin reactions occurred.

### Prediction of blood urea levels from cathodal buffer urea concentration

**Figure 2:**
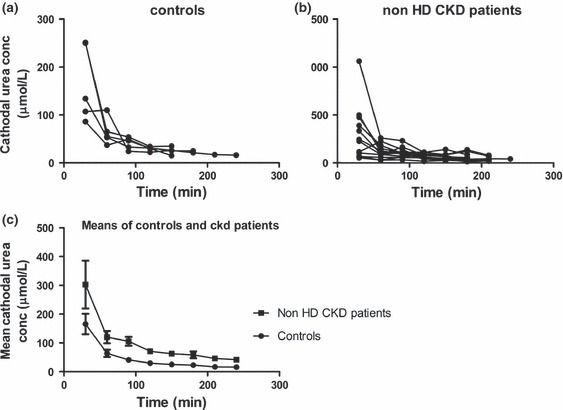
Evolution of cathodal urea concentration over time of iontophoresis. The urea concentration in the cathode buffer was initially higher in the first two collections, then diminished rapidly and became stable for the rest of the application (i.e. after 1 h of current application). This was true for both healthy controls (a) and non-haemodialysis chronic kidney disease patients (b). In (c), the means (+SD) of cathodal urea show a clear separation between patients and controls over the entire current application period (*P* = 0·03).

The concentration of urea in the 1·5 mL of cathodal buffer for each 30-min collection period showed the same pattern for controls and patients with CKD (Fig. 2). This was initially higher during the first two collections, that is, in the first hour, then subsequently fell and seemed to stabilise during the remaining duration of the experiment.

For controls and non-HD CKD patients, the cathodal urea concentration correlated significantly with plasma urea concentration. The strength of this correlation increased in the second hour (Pearson's coefficient, *r* increased from 0·67 at 1·5 h to 0·82 at 2 h) and continued to increase with time, attaining 0·89 at the end of a 3·5-h current application period. Table 2 summarises the correlation data between cathodal urea and plasma urea for patients with CKD and controls at successive sampling times.

**Table 2:** Summary of correlation data between iontophoretically extracted cathodal urea and plasma urea

Sampling time	0·5 h	1 h	1·5 h	2 h	2·5 h	3 h	3·5 h
*r*	0·69	0·52	0·67	0·82	0·84	0·82	0·89
*P*	0·002	0·03	0·003	<0·0001	<0·0001	0·0007	0·007
*R*^2^	0·48	0·26	0·45	0·68	0·71	0·66	0·80

Cathodal urea concentration was directly proportional to plasma urea concentration (0·03 ≥ *P* ≤ 0·0001), especially after 2 h or more of current application.

**Figure 3:**
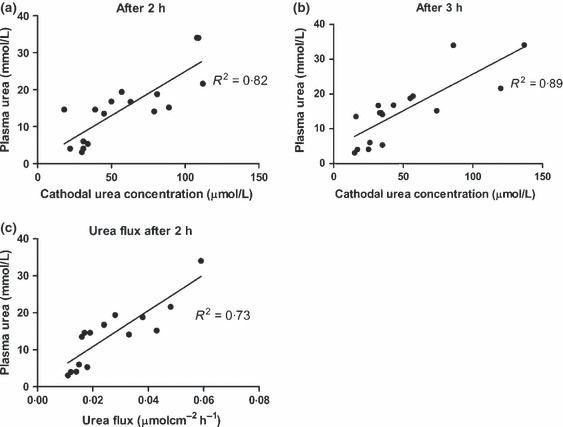
Linear regression graphs of cathodal urea concentration (a, b) and urea flux (c) versus plasma urea. Cathodal urea concentration and plasma urea were directly proportional after 2 h (a) and 3 h (b) of current application and reached statistical significance (0·0007 ≥ *P* ≤ 0·0001). These curves and the derived regression equations could therefore be used to estimate plasma urea concentration or as a surrogate for this measurement. The relationship is maintained with extracted urea expressed as flux (c).

Given the correlation between iontophoretically extracted and plasma urea (Table 2), an attempt was made to predict plasma urea concentrations from the cathodal concentrations. As the correlations become stronger after 2 h, the 2 and 3 h cathodal urea concentrations were used to predict plasma urea by regression analysis. A linear relationship was found between cathodal urea and plasma urea, defined by the equation, *y* = 0·24*x* + 1 [slope 95% CI 0·12–0·29] at 2 h and *y* = 0·21*x* + 4·63 [slope 95% CI 0·15–0·32] at 3 h, where *y* = plasma urea concentration (mM) and *x* = cathodal urea concentration (μM). Figure 3 depicts the linear regression curves for predicting plasma urea from cathodal urea concentration after 2 and 3 h of reverse iontophoresis, respectively. Plasma urea could be reliably predicted from cathodal urea concentrations or urea flux (amount of urea extracted per unit skin area per unit time) after 2 h or more of current application.

### Comparison of cathodal urea between patients and controls

**Figure 4:**
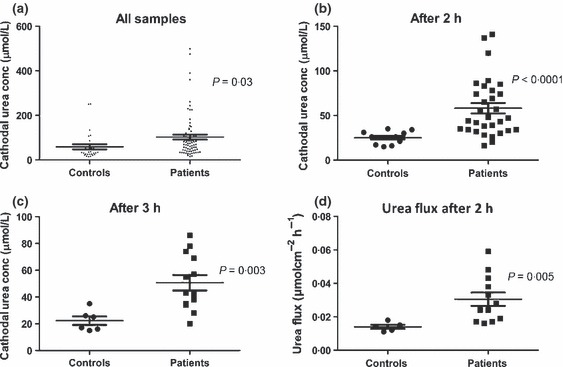
Comparison of cathodal urea concentration between patients and controls. Means plus spread of urea concentrations in patients were higher than those in controls (a). This segregation seemed even more apparent when the samples obtained from the first hour and a half were excluded. There was a significant difference between patients and controls after 2 h (b) and after 3 h (c). The separation was maintained when urea concentrations were converted to flux (μmol/cm^2^/h) (d).

The cathodal urea concentrations extracted from patients were significantly higher than those from controls (*P* = 0·03). Figure 4 depicts scatter plots of cathodal urea concentrations for controls and non-HD CKD patients overall (a), after 2 h (b) and after 3 h (c). The mean (+SD) cathodal urea after 2 h was 25·1 ± 6·8 μM [95% CI 20·8, 29·4] in controls and 57·5 ± 30·9 μM [95% CI 42·3, 76·7] in patients with CKD (*P* < 0·0001). After 3 h, mean cathodal urea was 22·3 ± 7·8 μM [95% CI 14·2, 30·5] in controls and 50·7 ± 7·8 μM [95% CI 38·1, 63·3] in patients (*P* = 0·003). Using a cathodal urea cut-off for CKD of 30 μM, the sensitivity of this test to distinguish patients with CKD from healthy controls after 2 h of iontophoresis was 83·3% [95% CI 62·68, 95·3] and the specificity 75·0% [95% CI 42·8, 94·5]. The positive predictive value was 87·0% [95% CI 66·4, 97·2] and the negative predictive value 69·2% [95% CI 38·6, 90·9].

### Tracking rapid changes in plasma urea during HD

**Figure 5:**
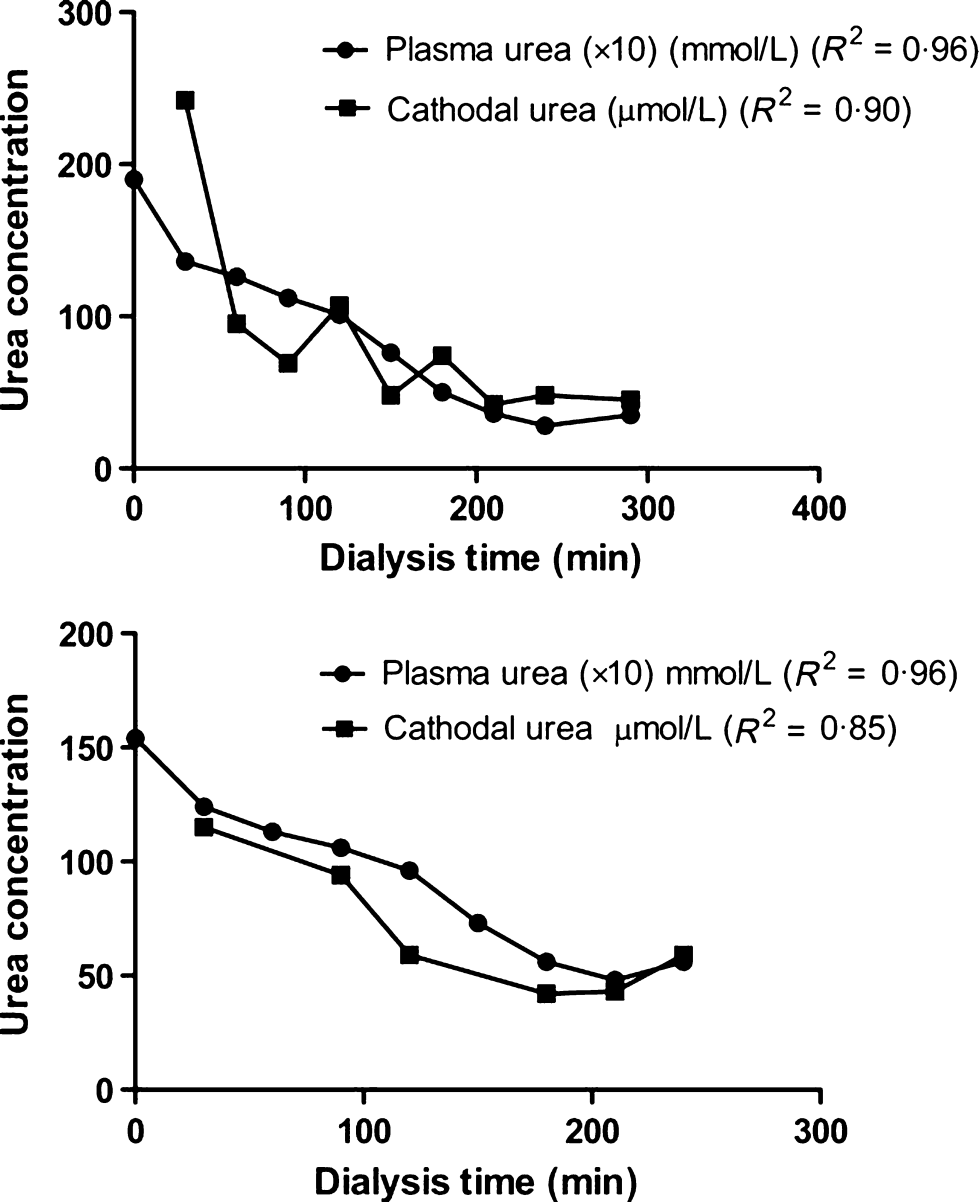
Decay of plasma urea and cathodal urea with time in two patients, during a haemodialysis (HD) session. Cathodal urea followed the same trend as plasma urea during the rapid changes induced by HD.

During HD, rapid clearances across the dialyser induce a sharp fall in plasma urea concentrations over the 4-h period, induced by the process of dialysis. In subjects studied during HD, plasma urea correlated significantly with cathodal urea (*r* = 0·64, *P* < 0·0001). When plasma urea concentration changes during HD were plotted against iontophoretically extracted urea (cathodal urea concentration) over the same period of time, the two methods of urea estimation showed a parallel single phase exponential decay as shown in the two patients in Fig. 5. This relationship strengthened further if the initial cathodal urea was excluded from the analysis. This indicates that iontophoretically extracted urea can track rapid changes in plasma urea levels.

## Discussion

### Safety and feasibility

This study demonstrates safety and tolerability of reverse iontophoresis (using a current of 250 μA) in healthy controls and patients with CKD for up to 5 h. Although other studies using larger currents (up to 800 μA) [23] have also reported no adverse events, this study defines the use of effective lower current intensities for extraction of measurable micromolar concentrations of urea at the cathode. These were sufficient to be analysed by the diacetylmonoxime method, which proved to be very accurate and precise, with a mean intra- and inter-assay variability of 8·7% and 3·6%, respectively. The development of a highly sensitive urea analysis method capable of being used in a routine biochemistry laboratory analyser will be necessary, however, to bridge the gap to a potential clinical utility of reverse iontophoresis for noninvasive diagnosis and monitoring of CKD.

### Predicting plasma urea and identifying patients with CKD from controls

This study confirms that iontophoretically extracted urea could distinguish between patients with CKD and healthy volunteers. Figure 2 shows clear separation, of the mean and standard deviation of iontophoretically extracted urea concentrations between patients and controls. The separation was more pronounced when the analysis excluded the initial reverse iontophoresis samples, collected in the first hour and a half (Fig. 3). This finding confirms that of other previous investigators in adult patients and controls [15].

Cathodal urea was mostly expressed as a concentration, similar to Degim *et al.* [24]. Urea extraction by reverse iontophoresis is often expressed as urea flux (μmol/cm^2^/h) [25]. However, as cathodal surface area, buffer volume and duration of current application were constant for each sample, the urea flux was directly proportional to the urea concentration. It was therefore logical to express extracted urea as concentration, as in routine clinical practice.

At 2 h of current application, cathodal urea was strongly correlated with plasma urea concentrations (Table 2). A simple linear regression equation could hence be used to predict plasma urea after 2 h of reverse iontophoresis. Wascotte *et al.* [14,15] found a similar relationship both *in vivo* and *in vitro*, although their current application only lasted 2 h. In our participants, longer current application seemed to improve the linearity, with the highest correlation coefficient (0·89) obtained after 3·5 h. Although it was not a primary aim of this study, we found that an arbitrary cathodal urea cut-off of 30 μM (based on the upper 95% CI of cathodal urea in controls after 2 h) gave a sensitivity of 83·3% and positive predictive value of 87% in segregating patients with CKD from controls. This demonstrates some promise in identifying patients with CKD in a given population. Further studies are required to corroborate this finding in larger numbers of subjects, especially among patients with intermediate GFRs (CKD stages 2 and 3), as the patients in this study had severe renal impairment (CKD stages 4 and 5).

### The ‘early lag’ phenomenon

The very high urea concentrations within the first hour of current application deserve further attention. The separation between patients and controls improved with time, with significant *P* values obtained after 2 and 3 h of current application. Our study lends supports to the minimum current application period of 2 h [15] and suggests than an even longer period might be better. The optimal duration for current application seems to be 3–3·5 h, although 2 h is likely to be sufficient if there are time constraints. The separation of the plasma values and iontophoretically extracted urea values may be explained by a urea reservoir in the dermal and subcutaneous tissues. A urea reservoir within the skin has been demonstrated [26]. The dynamic equilibration or steady state of the urea levels in plasma and extraplasmatic compartment is best achieved after at least 2 h. Whilst values in the first hour of the experiments approximate less the plasma values, their estimation may provide useful pathophysiological information of the extravascular accumulation of diffusible small metabolites and the burden of uraemic toxicity in cellular and interstitial compartments, not captured by plasma measurements alone. The biological significance of this ‘early lag’ phase merits further attention.

### Tracking rapid changes in urea levels during HD

Measurements during HD provided a natural interventional experiment where rapid changes in plasma values of urea can be induced to assess the sensitivity of the reverse iontophoresis technique. Cathodal urea concentrations in our patients decayed similarly to plasma urea concentrations during dialysis (Fig. 5). This indicates that the iontophoretically extracted urea can track real-time urea changes, creating further potential for intradialytic monitoring. HD efficacy in removing small water-soluble toxins is often assessed by plasma urea removal during dialysis [27]. This technique provides an opportunity for noninvasive real-time monitoring of dialytic urea removal, a potential novel application tool for assessing dialysis efficiency. Our findings confirm similar findings of one previous study using reverse iontophoresis on patients undergoing HD [15].

The main shortcoming of the reverse iontophoresis technique may be perceived as the need for a ‘warm up’ period of at least 1 h before useful sampling reflecting plasma values could commence. However, in certain clinical situations as in HD or when monitoring trends in acute settings, this may be clinically less significant. This drawback is offset by the noninvasive, well tolerated nature of the technique. A miniaturised, unobtrusive design is conceivable, such as with the Glucowatch® Biographer [12]. It may be worthwhile studying whether this ‘warm up’ period can be shortened by altering iontophoresis parameters like current, buffer formulation, temperature and analyte of interest. Also, the day to day reproducibility of the reverse iontophoresis procedure in the same individual, not tested in this study, will be worth addressing in future studies. In monitoring CKD, several other biochemical parameters are often measured simultaneously (e.g. creatinine, phosphate, calcium, bicarbonate, potassium and parathyroid hormone). Ionic uraemic retention solutes like potassium could be simultaneously monitored with urea by reverse iontophoresis [14,15], but occasional clinic visits may still be necessary to assess these other functions.

## Conclusion

Reverse iontophoresis is a noninvasive safe technique that can be reliably used to predict plasma urea in both patients with CKD and controls, and track changes of the metabolite during both steady states and rapid flux states. These findings indicate that studies aimed at adapting the technology for application in clinical settings and in the management of CKD merit further attention.

## Address

Department of Renal Medicine and Renal Research, Manchester Royal Infirmary, Manchester, UK (L. M. Ebah, I. Read, J. Morgan, P. Brenchley, S. Mitra); School of Biomedicine, University of Manchester, Manchester, UK (L. M. Ebah, P. Brenchley); Department of Clinical Biochemistry, Manchester Royal Infirmary, Manchester, UK (A. Sayce, C. Chaloner).
